# Cooperation between Prostaglandin E2 and Epidermal Growth Factor Receptor in Cancer Progression: A Dual Target for Cancer Therapy

**DOI:** 10.3390/cancers15082374

**Published:** 2023-04-19

**Authors:** Federica Finetti, Lucrezia Paradisi, Clizia Bernardi, Margherita Pannini, Lorenza Trabalzini

**Affiliations:** Department of Biotechnology, Chemistry and Pharmacy, University of Siena, 53100 Siena, Italy; lucrezia.paradisi@student.unisi.it (L.P.); clizia.bernardi@student.unisi.it (C.B.); margherita.pannini@student.unisi.it (M.P.)

**Keywords:** COX2, mPGES1, PGE2, EGFR, TKRs, cancer, tumor angiogenesis, tumor progression, intrinsic inflammation, extrinsic inflammation

## Abstract

**Simple Summary:**

Inflammation is the biological response of the body to damaging and toxic stimuli, and is a positive event that evolves with the resolution of critical events (acute inflammation). However, when the process becomes chronic it acquires pathological characteristics, and is associated with detrimental diseases such as cancer. It is recognized that prostaglandin E2 (PGE2) is one key lipid mediator involved in chronic inflammation, and is directly implicated in tumor development by regulating cancer cell growth and migration, apoptosis, epithelial–mesenchymal transition, metastasis, angiogenesis, and immune escape. The enzymes’ expression in PGE2 synthesis positively correlates with tumor progression and aggressiveness. This review describes the interplay between the PGE2 cascade and epidermal growth factor receptor that fuel cancer progression, and new therapeutic strategies that target these signaling pathways, to outline the importance of the modulation of the inflammatory process in cancer fighting.

**Abstract:**

It is recognized that prostaglandin E2 (PGE2) is one key lipid mediator involved in chronic inflammation, and it is directly implicated in tumor development by regulating cancer cell growth and migration, apoptosis, epithelial–mesenchymal transition, angiogenesis, and immune escape. In addition, the expression of the enzymes involved in PGE2 synthesis, cyclooxygenase 2 (COX-2) and microsomal prostaglandin E synthase 1 (mPGES1), positively correlates with tumor progression and aggressiveness, clearly indicating the crucial role of the entire pathway in cancer. Moreover, several lines of evidence suggest that the COX2/mPGES1/PGE2 inflammatory axis is involved in the modulation of epidermal growth factor receptor (EGFR) signaling to reinforce the oncogenic drive of EGFR activation. Similarly, EGFR activation promotes the induction of COX2/mPGES1 expression and PGE2 production. In this review, we describe the interplay between COX2/mPGES1/PGE2 and EGFR in cancer, and new therapeutic strategies that target this signaling pathway, to outline the importance of the modulation of the inflammatory process in cancer fighting.

## 1. Introduction

The inflammatory process is a complex biological response of the body to damaging and toxic stimuli, and is considered a positive event that evolves with the resolution of critical events (acute inflammation) [1]. However, when the process becomes chronic, it acquires pathological characteristics, and is associated with detrimental diseases grouped into the class of chronic inflammatory diseases. In addition, chronic inflammation is considered a critical factor able to promote cancer aggressiveness [2,3,4,5].

Cancer is one of the major causes of death in industrialized countries, and despite advancements in diagnosis and therapeutic approaches, remains a problem with great social and economic impact. Malignant transformation of normal cells may occur as a consequence of genetic and epigenetic alterations of cancer-related genes (oncogenes or oncosuppressor genes), and is associated with the disruption of key processes that are involved in the control of normal cell growth and tissue homeostasis. Other than genetic alterations of normal cells, the microenvironment surrounding transformed cells represents the drive of tumor development and progression [6,7]. Starting from Virchow in 1863, it became evident that inflammation, and mainly chronic inflammation, is associated with the majority of tumors, and supports their progression through the promotion of growth, migration, invasion, apoptotic escape, angiogenesis, and metabolic reprogramming of cancer cells [8]. It is accepted that microbial and viral infections, autoimmune diseases, and inflammatory conditions of different origin are triggers of chronic inflammation associated with cancer development. For example, *Helicobacter pylori* and the hepatitis C virus are associated with gastric cancer and hepatocellular carcinoma, respectively, inflammatory bowel disease is associated with colon cancer, and prostatitis may promote prostate cancer [3,5,8,9]. All these conditions are included in the definition of “extrinsic inflammation”, which includes all the events associated with an inflammatory milieu.

Moreover, the activation of pathways that promote the production of inflammatory mediators and the recruitment of inflammatory cells is also observed in tumors that are not directly related to surrounding inflammatory conditions [3,9]. It has been demonstrated that different genetic alterations of proto-oncogene (oncogene activation) may result in both cancer and inflammation. Abundant evidence has clearly demonstrated that the expression of the inflammation-related pathways is driven by the activation of different classes of oncogenes [2,3,8,9,10]. In this context, the definition of “intrinsic inflammation” describes the inflammatory process observed in cancer that is related to intrinsic characteristics of tumor cells and associated with the expression and induction of specific inflammatory pathways.

To further outline the fundamental role of inflammation in driving tumor progression, in 2011, Hanahan and Weinberg, who had previously published a famous manuscript that summarized six main common cancer hallmarks [11], expanded their considerations and inserted the inflammation process as an enabling characteristic of tumors [12], outlining how inflammation contributes to the appearance of multiple hallmark capabilities of cancer cells. The inflammatory milieu can promote cancer cell growth, migration, tumor angiogenesis, and metastasis by supplying bioactive molecules as growth factors, proangiogenic factors, extracellular-matrix-modifying enzymes, and proinflammatory mediators (such as cytokines, chemokines, and eicosanoids) [13,14,15,16]. The presence of inflammatory conditions in the tumor microenvironment drives tumor progression by activating different processes, such as the induction of genomic instability, altering gene expression as a consequence of epigenetic events, enhancing the proliferation and resistance to apoptosis of cancer cells, promoting cell motility, and inducing tumor angiogenesis and tissue remodeling, with the consequent promotion of tumor cell invasion and metastasis [3,9].

Among all the proinflammatory mediators, prostaglandins, mainly prostaglandin E2 (PGE2), appear to be a fundamental piece of the genesis and progression of cancer [17,18,19,20,21]. To corroborate the protumoral role of PGE2, several epidemiological studies have highlighted that the treatment with non-steroidal anti-inflammatory agents (NSAIDs), such as cyclooxygenase (COX) inhibitors, reduce the risk of developing certain cancers (i.e., colon and breast cancer) and the mortality related to these diseases [21]. 

Several molecular mechanisms have been proposed to justify the protumoral activity of PGE2. In vitro and in vivo studies demonstrate that PGE2 may induce epigenetic modifications that contribute to the growth and metastasis formation of breast and gastric cancer [22,23], promote miRNA modifications that contribute to modulate cancer cell growth and migration and tumor angiogenesis [24,25,26,27], and increases cancer cell growth, migration, and resistance to apoptosis through the modulation of several molecular pathways, such as JAK/STAT [28], PI3K/AKT [29], and RAS/Raf/MAPK signaling [30]. Moreover, PGE2 is involved in cancer immunomodulation, as previously reviewed [21].

In addition, a plethora of studies have reported the strong interplay between prostaglandins and receptors other than their own involved in tumor progression, outlining and suggesting the strong potential of inflammation in driving tumor aggressiveness [31,32,33,34].

In this review, we summarize the protumoral activity of PGE2 inflammatory pathways, mainly focusing on the interplay with the epidermal growth factor receptor (EGFR), and we show the recent advancement in the pharmacological modulation of these signaling pathways in cancer management.

## 2. Prostaglandin E2

PGE2 is now considered a promoter of tumor progression by boosting the appearance of several cancer hallmarks [21]. This notion has been established by experimental and epidemiological studies that reported increased expression of cyclooxygenase isoforms (COX-1 and COX-2) and microsomal prostaglandin E synthase (mPGES-1), as well as enhanced levels of PGE2, in several tumor types (colon, breast, prostate, and lung tumors) [17,18,19,21,35].

**Figure 1:**
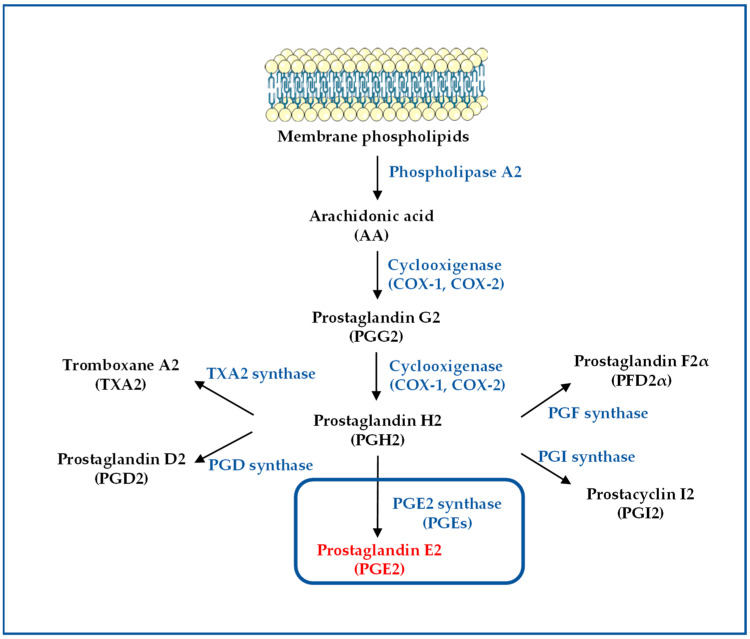
Biosynthesis of prostaglandins. Arachidonic acid (AA) is a phospholipid present in cell membranes and is liberated from the cellular membranes by cytoplasmic phospholipase A2 (PLA2). Free AA is converted to PGE2 through the COX pathway. AA is metabolized to the intermediate prostaglandin G2 (PGG2), which is then reduced to PGH2 by the peroxidase activity of COX. PGH2 is sequentially metabolized to PGE2 or other eicosanoids by specific synthases.

The biosynthesis of prostaglandins, i.e., the COX pathway, is an essential component of inflammatory responses, and may be commonly activated by several proinflammatory mediators, such as lipopolysaccharides (LPS) [36], cytokines [37], and xenobiotics [38,39]. PGs biosynthesis starts from arachidonic acid (AA), which is released by the action of various phospholipases, and is initially converted to an unstable prostaglandin G2 (PGG2), which is further reduced by the peroxidase activity of the same enzyme to a stable prostaglandin H2 (PGH2) (Figure 1). The enzymes that catalyze these steps are COX-1 and COX-2. COX-1 is constitutively expressed in many cell types, and is responsible for the generation of low levels of prostaglandins involved in body homeostasis. In contrast, COX-2 is not expressed in physiological conditions, but is induced by different stimuli, such as growth factors and cytokines [19,40,41].

Furthermore, the production of all PGs, such as prostaglandin D2 (PGD2), PGE2, prostaglandin F2α (PGF2α), prostacyclin (PGI2), and thromboxane A2 (TXA2), occurs by the action of different specific terminal synthases [42]. PGE2 is synthesized through the action of three different prostaglandin E2 synthases (PGESs). The PGES family includes cytosolic prostaglandin E synthase (cPGES) [43], microsomal PGES-1 (mPGES-1) [44], and microsomal PGES-2 (mPGES-2) [45], and each of them plays a different role in the synthesis of PGE2 (Figure 1). In cancer tissues, both COX-2 and mPGES-1 are overexpressed [46]. 

**Figure 2:**
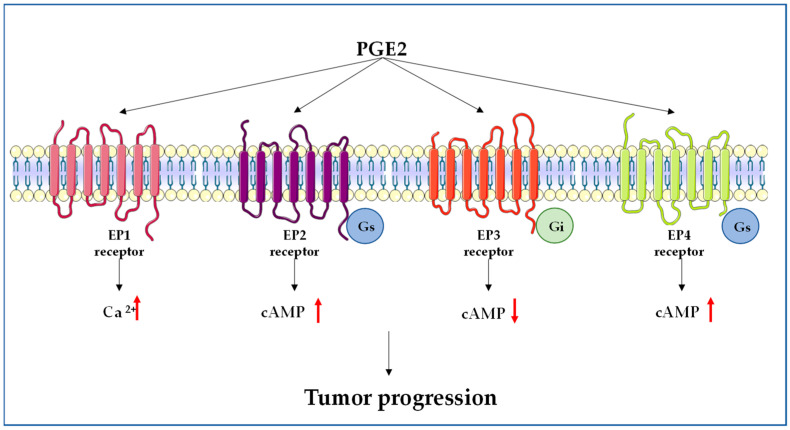
Schematic representation of EP receptors. PGE2 activity is mediated by the interaction with four G-protein-coupled receptors (GPCRs), namely, EP1–EP4 receptors. Each EP receptor possesses a distinct signaling pathway.

The biological actions of PGE2 are mediated by specific signaling pathways activated via various G-protein-coupled receptors, which include EP1, EP2, EP3, and EP4 receptors (Figure 2). The distribution and relative abundance of these receptors vary between different animal species and tissues [47]. Their signaling mechanism involves the G-protein-mediated activation of downstream targets via second messengers, such as cyclic AMP (cAMP), Ca^2+^, and inositol phosphates. More in detail, the activation of the EP1 receptor induces Ca^2+^ mobilization, whereas EP2 and EP4 receptor signaling is mediated by Gs protein and induces adenylyl cyclase (AC) activation and the increase in cAMP levels. EP3, which exists in multiple splicing isoforms, is coupled with Gi proteins, and inhibits AC, leading to a decrease in cAMP levels. Several in vitro and in vivo studies indicate that the specific activation of one of these receptors by PGE2 may promote cancer progression [48,49].

A high volume of data indicate that PGE2 promotes tumor progression, since both COX2 or mPGES1 upregulation, and/or high levels of PGE2, are linked to the development of several types of cancers [21,42,46,50,51]. 

In human cancer specimens, it has been observed that COX-2 and/or mPGES-1 are constitutively expressed in non-small cell lung cancer [52,53], colorectal cancer [54,55], breast cancer [56,57,58], prostate cancer [59,60], melanoma [61,62], and hepatocellular carcinoma [63,64], and contribute to tumor aggressiveness. Similar results have been reported in animal models, where overexpression of COX-2 and mPGES1 induces tumor formation, while its inhibition suppresses tumorigenesis or tumor progression [21]. For example, mPGES-1 is overexpressed in gastrointestinal-hamartoma-induced mice [65], increases the number of aberrant crypt foci at an early stage and increases tumor size in mice colorectal cancer [66], and promotes intestinal tumorigenesis in LPS-induced transgenic mice [67]; meanwhile, mPGES-1 deletion suppresses intestinal tumorigenesis in Apc(Min/+) mice [68]. Increased PGE2 synthesis was also reported in models of rat prostate cancers, and was linked to a worse prognosis [69]. The contribution of this signaling has been reported also in the immunogenic response to cancer, as extensively discussed in a previous review [21].

During cancer onset and progression, in addition to the dysregulation of the key enzymes involved in the biosynthesis of PGE2, it has been described that there is an aberrant expression and activation of EP receptors [47,50,70] (Figure 2). Extensive analysis of the role of EP receptors has been reported for colorectal cancer. In this type of tumor, the EP4 expression levels are upregulated during carcinogenesis, as reported in in vitro and in vivo models [71,72,73]. Watanabe et al. [74] demonstrated that the EP1 receptor is involved in the early stages of colon carcinogenesis, while EP3 receptor contributes to the later stages. However, Shoji et al. [75] showed that the expression levels of EP3 receptor are low in AOM-induced tumors, and that its deficiency increased the incidence and multiplicity of the tumor. Furthermore, it has been reported that the EP2 receptor plays a key role in tumorigenesis in the small intestine [76]. The loss of the EP2 receptor in ApcΔ716 compound mutant mice caused a reduction in the size and number of intestinal tumors. Furthermore, PGE2-EP2 receptor signaling promotes colon cancer cell migration, and amplifies the actions of COX-2 by increasing cAMP levels [77]. Considering the above-reported data, PGE2 is important in the development and progression of colorectal cancer. Still, there are several pieces of evidence that PGE2 may affect not only cancer cells, but also cells in the tumor microenvironment [21,78]. Moreover, in colorectal cancer, PGE2 is also involved in tumor immune evasion by suppressing the activity of immune cells (CD8+ T cells) and macrophages. PGE2 induces the expression of proteins involved in the suppression of the immune system, such as programmed death ligand 1 (PD-1), through the activation of the EP4 receptor, and by reducing the number of CD8+ T cells [79].

Several recent studies have focused on the potential role of EP receptor signaling in other types of cancers. For example, EP1, EP2, and EP4 receptor expression is increased while EP3 receptor expression is reduced in prostate cancer tissues, and EP1 receptor expression seems to be positively associated with tumor grade and TNM stage [80,81]. 

Moreover, recent studies suggest that the effects of PGE2 on tumor progression are also related to the effects of this prostaglandin on the tumor microenvironment, and actively trigger tumor immune evasion, influencing patient survival [21,82,83,84]. Furthermore, in vivo experiments on different tumor-based animal models indicate that the EP4 receptor may be involved in immune cell activity modulation during tumor growth [58,85,86,87,88,89]. However, the mechanisms by which the pharmacological modulation of EP4 signaling may be useful in cancer immunotherapy have not yet been clearly demonstrated.

In addition to the above-reported data, PGE2 may also elicit its protumoral effects through the activation of non-canonical pathways, in which the PGE2-mediated effects are linked to the activation of receptors different from its own. In the past few years, the interplay between PGE2 and several tyrosine kinase receptors has been extensively studied, with particular attention given to EGFR. Firstly, in 2002, it was demonstrated that PGE2 promotes EGFR phosphorylation and triggers the ERK2 signaling pathway in normal gastric epithelial and colon cancer cell lines. Inhibition of EGFR with selective inhibitors blocks PGE2-induced ERK2 activation, c-fos mRNA expression, and cell proliferation, indicating the importance of this signaling pathway in cell biology [33]. In 2003, Buchanan and co-workers demonstrated that PGE2 induces the migration and invasion of colorectal cancer cells through rapid transactivation and phosphorylation of EGFR [90]. They demonstrated that, in cellular models, PGE2-induced EGFR phosphorylation occurs through the activation of an intracellular pathway (Src-mediated activation) rather than through the release of an extracellular epidermal growth factor-like ligand. They also reported that EGFR transactivation was present in malignant human colorectal samples [90]. In the same year, Pai et al. showed that PGE2-increased colon cancer cell invasiveness is consequent to the activation of the EGFR-c-Met-R-β-catenin-uPAR signaling pathway [32], confirming the existence of PGE2 non-canonical pathways. Similarly, in squamous cell carcinoma, PGE2 promotes tumor cell growth and invasion by the activation of the EP2 receptor, which in turn promotes EGFR transactivation via protein kinase A (PKA) and cSrc activation [91]. In the following years, a high volume of scientific evidence suggests that TKR activation may represent an important oncogenic signal induced by PGE2. In fact, EGFR transactivation induced by PGE2 has been observed in many tumor types, such as endometrial adenocarcinomas [92], gastric cancer [93], and prostate cancer [94]. In addition, EGFR transactivation has been reported for EP1 [95], EP2 [92], and EP4 [96], increasing the complexity of EP receptor signaling pathways.

**Figure 3:**
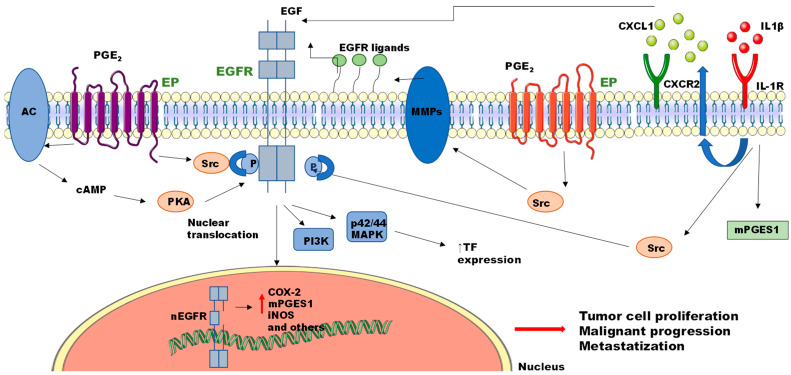
Crosstalk between PGE2 and EGFR. PGE2 promotes EGFR phosphorylation and internalization through the activation of different signaling pathways. PGE2 promotes EGFR phosphorylation either by EP-mediated Src or PKA activation, or by inducing the release of EGFR ligands (MMPs activation). Similarly, IL-1β signaling promotes EGFR activation through the increased production of CXCL1, which in turn activates EGFR through CXCR2, or by a Src-mediated mechanism. AC = adenylate cyclase; PGE2 = prostaglandin E2; EP = prostaglandin E2 receptor; cAMP = cyclic adenosine monophosphate; PKA = protein kinase A; Src = SRC proto-oncogene; EGFR = epidermal growth factor receptor; nEGFR = nuclear EGFR; EGF = epidermal growth factor; PI3K = phosphatidylinositol 4,5-bisphosphate 3-kinase; p42/44 MAPK = p42/44 mitogen-activated protein kinases; TF = tissue factor; MMPs = matrix metalloproteinases; IL-1β = interleukin 1β; CXCR2 = CXC motif chemokine receptor 2; CXCL1 = C-X-C motif chemokine ligand 1; COX-2 = cyclooxygenase 2; mPGES1 = microsomal prostaglandin E synthase 1; iNOS = inducible nitric oxide synthase.

Moreover, PGE2-induced EGFR activation is not limited to intracellular phosphorylation of the receptor, but may also be due to the extracellular mobilization of EGFR ligands [96,97,98]. Oshima and co-workers demonstrated that, in mouse models of gastric cancer, the expression levels of EGFR ligands (i.e., epiregulin, amphiregulin, heparin-binding EGF-like growth factor, and betacellulin), and metalloproteinases are increased in a PGE2-pathway-dependent manner. Indeed, metalloproteases (MMPs) can activate EGFR by the ectodomain shedding of EGFR ligands, dependent on the EP4 receptor [96]. Similarly, it has been demonstrated that PGE2, through EP3 receptor activation, promotes EGFR phosphorylation and its nuclear translocation through the induction of EGFR ligands in lung cancer cell models [97]. A schematic representation of PGE2/EGFR crosstalk is reported in Figure 3.

In summary, a vast amount of experimental data confirm that PGE2 promotes EGFR activation, albeit via different mechanisms. Depending on experimental conditions and cellular models, there are two main mechanisms by which PGE2 may affect EGFR activity: (1) mobilization of intracellular pathways (such as Src or PKA) that promote direct intracellular tyrosine phosphorylation of EGFR; (2) PGE2-induced activation of intracellular signaling that activates metalloproteinases, which in turn induce EGFR activation after EGFR ligand shedding from the plasma membrane (Figure 3).

In addition, it is important to outline that the link between PGE2 and EGFR was also observed in cellular and in vivo models of cancers where endogenous PGE2 production is abrogated by COX2 or mPGES1 inhibition. Pharmacological inhibition or genetic suppression of mPGES1 inhibits EGFR phosphorylation both in human epidermoid carcinoma cells and in in vivo animal models of cancer, with a consequent reduction in tumor growth and inhibition of angiogenesis [99].

The promotion of the activity of oncogenes, such as EGFR, is very important in cancer cells, where TKR phosphorylation induces the activation of several signaling pathways, such as the MAPK, PI3K/Akt, STAT, and PLC signaling pathways, that lead to cell growth, differentiation, migration, and survival. 

## 3. Epidermal Growth Factor Receptor, Cancer, and Intrinsic Inflammation

The ErbB family contains four proteins structurally and functionally related to the first discovered member EGFR (ErbB1), and involved in the pathogenesis and progression of numerous tumor types [100]. 

The ErbB proteins are normally expressed in different cell types, such as epithelial, mesenchymal, and neuronal cells, where they exert physiological roles. All four ErbB family members are tyrosine kinase receptors. They consist of an extracellular domain for ligand binding, a hydrophobic transmembrane region, and an intracellular region for signal transduction, with a conserved tyrosine kinase domain [100,101]. After EGFR activation by a specific ligand, multiple adaptors and signaling molecules are docked to the phosphorylated site and generate diverse intracellular responses. For example, the PI3K-Akt and RAS-ERK pathways [100,101]. 

EGFR overexpression or aberrant activation has been found in several tumors and drives tumor progression, inducing cancer cell growth, migration, invasion, and metastasis [102,103]. EGFR overexpression is an unfavorable prognostic marker in lung cancer [104], squamous cell carcinoma (SCC) [105], colorectal cancer [106,107], and others. Inhibition of EGFR activation through monoclonal antibodies (such as cetuximab and panitumumab) or small tyrosine kinase inhibitors (i.e., erlotinib and gefitinib, afatinib, and osimertinib) is an important pharmacological approach in several tumors, including those of the lung, pancreas, and colon, where the EGFR receptor is highly expressed, constitutively activated, or mutated [108,109,110]. 

Despite the initial favorable response to these treatments, most patients become resistant to the therapy [111]. Potential mechanisms involved in the appearance of resistance to EGFR inhibition include EGFR amplifications or mutations in the kinase domain [112,113]; constitutive activation of signaling pathways independent of EGFR, such as the coactivation of multiple TKRs [111]; promotion of the ERK5 pathway [114], or a pathway downstream of EGFR, such as the Ras/ERK1/2 and PI3K pathway [110,115,116]; and the activation of drug efflux mechanisms [113]. New studies are aimed at circumventing the resistance to EGFR inhibition by using combination therapies that engage EGFR inhibitors together with other treatment modalities targeting downstream EGFR signals.

In this scenario, extensive studies on the interplay between EGFR signaling and inflammation appear very important. Much evidence supports the idea that EGFR signaling and inflammation may be closely interconnected with each other. Both external inflammatory stimuli (extrinsic inflammation) and tumor-cell-derived inflammatory mediators (intrinsic inflammation) produced by tumor cells promote EGFR phosphorylation or the activation of EGFR molecular signaling. 

For example, interleukin-1 beta (IL-1β), a well-known mediator of chronic inflammation, has been identified as a salivary biomarker for oral squamous cell carcinoma [117], and increased IL-1β levels have been related to the increased severity of oral malignant transformation in in vivo models [118]. In addition, oral squamous carcinoma cell lines secrete high levels of IL-1β, which promotes tumor growth in an autocrine manner (intrinsic inflammation). Lee and co-workers showed that IL-1β can modulate EGFR activation through IL-1β-dependent CXCL1 expression, which promotes carcinogenesis [119]. Similarly, it has been demonstrated that IL-1β promotes tissue factor (TF) production in adenocarcinoma cell lines through the induction of Src-mediated EGFR phosphorylation [120]. To outline the importance of intrinsic inflammation in EGFR activation, it has also been demonstrated that IL-1β promotes EGFR phosphorylation by increasing PGE2 levels through the induction of mPGES1 expression [99] (see Figure 3).

Similarly, leukotrienes, derived from the arachidonic acid cascade through the action of lipoxygenase (LOX), may play a role in cancer progression [121]. As for other inflammatory stimuli, the interplay between COX and LOX pathways has been described, and the two distinct eicosanoid groups are likely to be able to mutually foster each other’s effects [122,123]. Leukotrienes, particularly leukotriene B4 (LTB4) and leukotriene D4 (LTD4), have been reported to be highly involved in EGFR signaling, and some experimental data indicate that they are able to induce EGFR transactivation [124,125,126].

As described above, a particular loop has been reported between EGFR and the COX2/mPGES1/PGE2 axis. Both exogenous PGE2 (produced by the tumor microenvironment) and endogenous PGE2 (produced by the tumor cell itself) promote EGFR phosphorylation through direct or indirect mechanisms. On the other hand, EGFR activation alone promotes the upregulation of both COX2 and mPGES1 enzymes associated with increased PGE2 production and enhanced tumor aggressiveness [127,128,129]. In head and neck squamous cell carcinoma, EGF regulates metastasis through the induction of angiopoietin-like 4 (ANGPTL4), which was inhibited after genetic or pharmacological inhibition of COX-2 [130]. Similarly, COX2 regulates ovarian cancer cell invasion [131] and colorectal cancer cell migration induced by EGF [132].

In addition, PGE2-induced EGFR activation, through different EP receptors, may be responsible for the development of resistance to anti-EGFR therapies in some cancer patients [47].

On the other hand, it has been also demonstrated that in several tumor cell lines (SCC, colon, and lung), EGFR phosphorylation induces mPGES-1 upregulation through the activation of ERK1/2 and Egr-1 signaling, and that this enzyme drives the protumoral activity of EGFR [127]. In fact, in a vicious circle, the inhibition of the COX2/mPGES1/PGE2 pathway reduced the protumoral effects of EGFR activation [99]. In this light, dual inhibition of EGFR activation and PGE2 production may be a strategy to inhibit tumor progression and overcome EGFR therapy resistance. 

## 4. PGE2 and EGFR as a Dual Target for Cancer Therapy

Several preclinical studies report the antitumoral activities of compounds, of natural or chemical origin, that act as dual inhibitors of EGFR and the COX2/mPGES1/PGE2 axis. For example, berberine, an isoquinoline alkaloid present in different species of plants, inhibits intestinal tumor development through the downregulation of Wnt, EGFR signaling pathways, and COX-2 expression [133]. Similarly, avenanthramides, isolated from *Avena sativa*, helioxantyne, from *Taiwania cryptomerioides Hayata,* and FAG, a 2-O-a-L-rhamnopyranosyl-hexacosanoate-b-D-glucopyranosyl ester from *Ficus bengalensis*, are natural EGFR inhibitors and reduce EGFR phosphorylation and COX-2 expression in tumoral cells and macrophages [128,134,135].

The efficacy of EGFR/COX-2 dual inhibition has been reported in several cancer cell models and animal models of different tumor types. In 2007, it has been reported that the use of NS-398, a selective COX-2 inhibitor, and AG1478, an EGFR inhibitor, reduced the migration of colorectal cancer cells. The authors suggested combining non-steroidal anti-inflammatory drugs with EGFR antagonists for future use in the clinic [136]. Due to these and similar observations, Qian et al. reported that, in in vitro and in vivo models of oral squamous cell carcinoma, the administration of low concentrations of cetuximab and celecoxib reduces the proliferation, migration, and invasion of cancer cells and decreases PEG2 levels. They showed that the combination of these drugs significantly induces apoptosis and reduces the phosphorylation of EGFR, PI3K, and Akt, which may contribute to the inhibition of tumor growth [137]. Li et al. demonstrated that a combination of erlotinib and celecoxib inhibits tumor growth of NSCLC (non-squamous cell lung carcinoma) in vitro and in vivo, with synergistic effects [138]. Importantly, they found that the combination of celecoxib and erlotinib leads to synergistic cell death only in EGFR-mutated cell lines harboring EGFR exon 19 deletions [138]. Similarly, it has been reported that, in human patients, high serum COX-2 levels may correlate with EGFR mutations, and that the efficacy of combined celecoxib and gefitinib is significantly greater in NSCLC cells with EGFR mutations than in wild-type NSCLC cells [139]. 

Recently, a similar approach has been proposed for prostate cancer, and it has been demonstrated that, in prostate cellular models, celecoxib reduces cell growth, induces apoptosis, and promotes EGFR degradation. In addition, celecoxib, in association with cetuximab, reduced the invasive phenotype of CRPC (castration-resistant prostate cancer) cells by modulating NF-kB activity and tumor growth in mice xenografts [140].

Despite encouraging preclinical data, clinical trials, in which the combination of COX-2 specific inhibitors (celecoxib and apricoxib) with EGFR inhibitors (erlotinib) was tested, have shown poor activity [141]. However, Gitlitz and co-workers reported that, in a selected patient population in which urinary prostaglandin M (PGE-M) was modulated in response to COX-2 inhibitor, the primary endpoint of the study was not met, with no difference between patients treated with apricoxib and erlotinib. Nevertheless, in a subset analysis of patients aged 65 years or younger, the combination of the drugs demonstrated a statistically significant benefit compared with placebo/erlotinib groups. [141]. The need for patient selection was also demonstrated by Reckamp and colleagues, who showed that the combination of erlotinib and celecoxib did not improve outcomes in an unselected population, but selection by elevated baseline PGEM led to an increase in progression-free survival (PFS) with the celecoxib combination. They also outlined that patients with EGFR wild-type status may benefit from the combination [142].

In a recent meta-analysis, the authors showed that celecoxib combined with palliative therapy is not able to improve patient survival or the local control of the tumor. However, they reported that EGFR wild-type patients had a prolonged PFS with celecoxib-combined therapy [143]. These observations suggest that despite the fact that initial clinical trials evaluating the efficacy of COX-2/EGFR dual inhibition were not successful, further studies are needed to take into account the differences in patient stratification (i.e., prostaglandin metabolite monitoring, EGFR status characterization).

Furthermore, cancer characterized by resistance to EGFR therapy may represent a possible application of EGFR/COX2 inhibition therapy. Xiao et al. demonstrated that COX2-TXA2 signaling prevents apoptosis and promotes gefitinib resistance in NSCLC. The combination of celecoxib, TOPK inhibitor pantoprazole, and gefitinib resulted in the ability to induce apoptosis in gefitinib-resistant cells and to inhibit tumor growth in vitro and in vivo [144]. Similarly, the clinical use of osimertinib, a third-generation irreversible EGFR inhibitor with important benefits for EGFR-mutated patients, may be limited by the acquisition of resistance. Han and colleagues demonstrated using in vitro and in vivo experiments that aspirin sensitizes osimertinib-resistant NSCLC cells to osimertinib by promoting apoptosis, providing evidence for the use of the combination of aspirin with osimertinib to overcome resistance in NSCLC patients [145]. The activity of aspirin in tumors resistant to targeted therapies has also been shown in other models, where it increased the sensitivity of resistant tumors to targeted drugs and significantly delayed the emergence of acquired resistance [146].

## 5. Conclusions

In summary, chronic inflammation and cancer appear as closely related diseases, and share important signaling pathways and molecules that act in synergy with each other. Inflammation has been recognized as an important hallmark of cancer, and ultimately serves to support tumor development. How and, primarily, why cancer onset and progression need to use inflammatory pathways is not understood, but it is now a fact.

Therefore, pharmacological approaches aimed to fight tumor progression through the inhibition of inflammation are under investigation, and strongly suggest that improving the combination of therapies that inhibit pathological inflammation and stimulate the antitumor response may be a successful strategy for the treatment of patients with cancer. Although the strong association between the COX-2/mPGES-1/PGE2 pathway and EGFR and its molecular signals in cancer is widely reported, more in-depth studies are necessary to better understand the physiopathological significance and the potential for cancer therapy.
